# Supplementary material for: Sustainable and conventional banking in Europe
Source: PLoS One. 2020 Feb 20;15(2):e0229420. doi: 10.1371/journal.pone.0229420 (PMC7032717; doi:10.1371/journal.pone.0229420)
Supplement: Attachment — Submitted filename: Response to Editor and Reviewers.docx [file pone.0229420.s002.docx]

**Manuscript ID PONE-D-19-27736**

December 18, 2019

Dear Editor,

We have reviewed the manuscript according to the three reviewers’ suggestions. We would like to acknowledge their reviews, which undoubtedly have allowed us to improve the work. We have tried to consider and answer all the proposed questions. In this way, we hope to meet all your expectations and, of course, we are at your disposal for any other comments, which you deem appropriate to be included in our manuscript.

In the following paragraphs, we are going to show the comments we have received (in italics) and the changes we have done in relation to them:

- *“The data of BBVA correspond to Spain whilst the rest of entities provide international data. Therefore, data from BBVA must be completed in order to include international information”* (Reviewer 2 and Editor).
  - Initial data of BBVA have been changed to international data.
- *“Regarding the study period I think it is reduced and could have been extended further. Only three years are studied from 2015 to 2017. When the year 2018 could have been incorporated, which already provides financial data of the entities object of this study”* (Reviewer 1); *“…try to expand your dataset to 2018”* (Editor).
  - We have added the year 2018 in the study.
- *“… the time horizon of study is not very representative. At work it is not justified because the horizon under study from 2015 to 2017 is selected. And the relevance of this choice”* (Reviewer 1).
  - First, we have expanded the period from 2015 to 2018. In order to justify the selected period, we have included the following sentence at the end of the first paragraph in Section 2 (Materials and methods): “By considering that European ethical banks are relatively new banks (which implies that they have experienced their greatest growth in recent years) and since our aim is to analyse the current situation and to compare the relative figures between conventional and ethical banking, the elected period was the past four years”·
- *“Regarding the methodology used, although valid for the work, another type of statistical methodology could have been used that would give more value to the work presented”* (Reviewer 1). *“I would like to add that your current method does not allow to appropriately test your hypotheses. That is because the ANOVA pools data ending up with a cross-section. Therefore, there are a number of alternative explanations for your results that simply cannot be ruled out. The differences that you find could be attributed to the ethical attributes of the bank or any omitted variables that you left out the analysis. For more information on the validity of cross-sectional inferences, please see Spector, P.E. (2019). Do not cross me: optimizing the use of cross-sectional designs. Journal of business and psychology, 34(2), 125-137. I would suggest that you exploit the panel nature of your data and use panel fixed effects regression analysis to identify significant differences between conventional and non-conventional banks (with a dummy variable). This way, you can be more confident that the results are not driven by other characteristics or specific to particular point in time. You could leave the ANOVA test as a robustness analysis”* (Editor).
  - We have considered a dummy variable that takes the value 1 for ethical bank and 0 for conventional bank. To study the relationships between the variables considered in the statistical test and the banking models, we have performed individual probit regressions (see Table 8 in the article). First, as usual, we analysed the Pearson correlations by checking the high correlations between the variables. Thus, we decided, as usual in research, to perform individual models with each individual variable (Reverte, 2009; Valls & Abad, 2018), since multicollinearity prevented us from considering the variables together in a unique model. In all of them, we use ROE as a control variable, since ROE did not show significant correlations with the studied variables. You can see the correlation matrix below (this matrix is not reported in the paper due to its current extension and since we think that, in this case, it does not add value to the article).

| **Pearson correlations** | | | | | | | | | | |
| --- | --- | --- | --- | --- | --- | --- | --- | --- | --- | --- |
|  | ROE | TierCap | Indeteb | Guarant | Coverag | LoanAss | FundAss | VarLoan | VarFund | VarAss |
| TierCap | **-0.2481**  (0.0171) |  |  |  |  |  |  |  |  |  |
| Indeteb | -0.1514  (0.1498) | -0.0653  (0.5364) |  |  |  |  |  |  |  |  |
| Guarant | **0.2436**  (0.0193) | **0.2151**  (0.0394) | **-0.7233**  (0.0000) |  |  |  |  |  |  |  |
| Coverag | **0.2339**  (0.0248) | **0.2134**  (0.0411) | **-0.7556**  (0.0000 | **0.9982**  (0.0000) |  |  |  |  |  |  |
| LoanAss | **0.3646**  (0.0004) | -0.1969  (0.0599) | **-0.2795**  (0.0070) | **0.2478**  (0.0172) | **0.2566**  (0.0135) |  |  |  |  |  |
| FundAss | 0.0740  (0.4831) | -0.0723  (0.4934) | **-0.6009**  (0.0000) | **0.2655**  (0.0105) | **0.3022**  (0.0034) | **0.4503**  (0.0000) |  |  |  |  |
| VarLoan | 0.2390  (0.2720) | -0.3950  (0.0621) | **-0.6011**  (0.0024) | **0.5314**  (0.0091) | **0.5424**  (0.0075) | **0.5868**  (0.0032) | **0.6685**  (0.0005) |  |  |  |
| VarFund | 0.1997  (0.3609) | -0.3370  (0.1158) | -0.3469  (0.1049) | 0.3624  (0.0893) | 0.3663  (0.0856) | 0.2430  (0.2638) | **0.5571**  (0.0058) | **0.8473**  (0.0000) |  |  |
| VarAss | 0.2425  (0.2649) | -0.3030  (0.1599) | **-0.4977**  (0.0157) | **0.5022**  (0.0146) | **0.5088**  (0.0132) | 0.3443  (0.1076) | **0.5354**  (0.0085) | **0.8410**  (0.0000) | **0.9249**  (0.0000) |  |
| VarInc | 0.1930  (0.3775) | -0.4205  (0.5840) | **-0.6101**  (0.0020) | **0.6096**  (0.0020) | **0.6188**  (0.0016) | 0.3842  (0.0703) | **0.5722**  (0.0043) | **0.8209**  (0.0000) | **0.8026**  (0.0000) | **0.8014**  (0.0000) |

- We have considered a cross-sectional design since the dummy variable is constant over time for each bank. The effect of the dummy variable would be absorbed as a part not differentiable of fixed effect. Therefore, it is not possible to consider fixed effect.

Moreover, cross-sectional design is appropriate when an exploratory research is conducted and when it is unknown if two variables covary (Spector, 2019; p.133). In fact, “cross-sectional method should be the method of choice in cases where we need to establish that variables are related…” (Spector, 2019; p.136), and “Often overlooked is that the cross-sectional design can tell us much that is of value and that the longitudinal design is not necessarily superior in providing evidence for causation” (Spector, 2019; p. 125). In effect, it is usual that research shows not only the longitudinal study, when it is possible, but also the cross-sectional results (Lin & al., 2019; Ward & Forker, 2017; Joecks et al. 2013). This indicates the value of such studies.

At the end of Section 2 (Materials and methods), we have added the following paragraph: “Finally, we have considered a dummy variable, which takes the value 1 if the bank is ethical and 0 if it is conventional. To check the robustness of the obtained results, we have performed probit regressions between each of the analyzed variables in the previous statistical test and the dummy variable. In all of them, ROE ratio has been considered as a control variable”.

We have added the Section “3.5. Probit regressions” and Table 9 (see below). The content of Section 3.5. is the following:

“The probit regressions between the banking model and the individual explanatory considered variables show similar results to the statistical test. Tier 1 is not related to banking model, but the other variables are significant at 1% significance level. Ethical banks are less indebted and have better guarantee and coverage ratio. They are more linked to real economy with greater loans to assets and funding to assets ratios. And, the growth is positively related to ethical banks. The joint consideration of all variables has not been possible due to the high correlation between the financial parameters”.

| **Table 9.** Probit regressions between banking model and the individual explanatory variables. | | | | | | | | | | |
| --- | --- | --- | --- | --- | --- | --- | --- | --- | --- | --- |
|  | Tier 1 | Indetebness | Guarantee | Coverage | Loans/Assets | Funding/Assets | Var.Loans | Var.Funding | Var.Assets | Var.Income |
| Constant | 0.3666  (0.604) | 2.8638^***^  (0.000) | -43.2372^***^  (0.000) | -3.1618^***^  (0.000) | -3.4145^***^  (0.000) | -6.7201^***^  (0.000) | -2.2852^**^  (0.045) | -0.5626  (0.388) | -0.0243  (0.975) | -0.0414  (0.945) |
| ROE | 0.0135  (0.579) | 0.0025  (0.928) | 0.0099  (0.715) | 0.0091  (0.737) | -0.0497^*^  (0.094) | 0.0739^*^  (0.051) | 0.1707  (0.195) | -0.0973  (0.434) | -0.1413  (0.361) | -0.0567  (0.583) |
| Variable | -0.0178  (0.674) | -0.1973^***^  (0.000) | 40.2671^***^  (0.000) | 46.2339^***^  (0.000) | 6.2528^***^  (0.000) | 9.4582^***^  (0.000) | 0.1210^***^  (0.004) | 0.1035^***^  (0.000) | 0.1474^***^  (0.000) | 0.0981^***^  (0.000) |
| Observations | 92 | 92 | 92 | 92 | 92 | 92 | 23 | 23 | 23 | 23 |
| Pseudo *R*^2^ | 0.0047 | 0.2427 | 0.2720 | 0.2705 | 0.2216 | 0.5522 | 0.6725 | 0.6331 | 0.8003 | 0.5643 |
| McFadden’s Adjust *R*^2^ | -0.043 | 0.195 | 0.224 | 0.223 | 0.174 | 0.505 | 0.482 | 0.443 | 0.610 | 0.374 |
| Nagelkerke *R*^2^ | 0.0085 | 0.3791 | 0.4170 | 0.4150 | 0.3510 | 0.7114 | 0.8070 | 0.7774 | 0.8928 | 0.7217 |
| Wald chi2 | 0.58  (0.7489) | 26.33^***^  (0.0000) | 29.59^***^  (0.0001) | 29.23^***^  (0.0000) | 32.94^***^  (0.0000) | 69.22^***^  (0.0000) | 8.79^**^  (0.0124) | 15.44^***^  (0.0004) | 19.13  (0.0001) | 17.63^***^  (0.0001) |
| Hosmer-Lemeshow chi2 | 18.20  (0.0198) | 15.68  (0.0472) | 14.85  (0.0621) | 14.99  (0.0594) | 9.04  (0.3390) | 13.78  (0.0876) | 1.54  (0.9920) | 6.75  (0.5640) | 0.56  (0.9998) | 2.63  (0.9552) |
| Percent concordant | 56.52 | 70.65 | 72.83 | 72.83 | 67.39 | 80.43 | 91.30 | 86.96 | 91.30 | 78.26 |
| ^***^ and ^**^ indicate less than 1% significance level and less than 5%, respectively.  *p*-values are shown into brackets.  According to Wald test *p*-value should be less than 0.05 and according to Hosmer-Lemeshow test *p*-value should be more than 0.05.  Source: Calculated by the authors with Stata14. | | | | | | | | | | |

- *“(In the abstract) it is stated that “The results reveal that ethical banking is growing more than conventional banking and it presents greater liquidity and solvency, although, in general terms, its profitability is lower”. Additionally, in Section 3.3, it is stated that “Regarding to ROA and ROE, we do not find a common pattern in conventional banks and different in Triodos”. Consequently, I propose that, in the abstract, authors should state: “The results reveal that ethical banking is growing more than conventional banking and it presents greater liquidity and solvency, although, in general terms, its profitability is not higher”* (Reviewer 3).
  - It has been done.
- *“(In the abstract) Authors should mention the means difference test and the analysis of variance”* (Reviewer 3)
  - We have added the following sentence in the abstract: “To do this, we apply a financial and economic analysis to the period from 2015 to 2018, the means difference test and analysis of variance on an array of financial ratios and, finally, probit regressions”.
- *“It would be convenient to present a table with the main differences between conventional and ethical banking”* (Reviewer 2).
  - We have included the following sentence in the Introduction: “Table 1 shows the main differences between ethical and conventional banking”.

| **Table 1.** Main differences between ethical and conventional banking | | |
| --- | --- | --- |
| **Feature** | **Ethical banking** | **Conventional banking** |
| Objective | Triple bottom line: social, environmental and economic | Profit-maximizing to reward shareholders |
| Profit | A means to and end | An end in itself |
| Investment object | Only on real economy | Speculative transactions |
| Investment criteria | Positive screen (environment, employment creation, culture, etc.) and negative screen (armament, polluting companies, child exploitation, etc.) | Profitability and risk |
| Loan policy | Avoid financial exclusion | Exclusion of specific segments of the society |
| Transparency | Total transparency of information and allocation of assets | Lack of transparency |
| Geographical distribution | Low number of branches | High number of branches |
| Source: Made by authors. | | |

- *“(In the Introduction) In the fifth paragraph, it is stated: “First, we van highlight the distrust…”. It is clear that there is a typo which must be corrected: “First, we can highlight the distrust…”* (Reviewer 3).
  - It has been corrected.
- *“(In the Introduction) Table 1 should provide the information corresponding to 2016, in line with the rest of the tables”* (Reviewer 3).
  - It has been done. We have also included 2018. (The former Table 1 is now Table 2)
- *“I miss a section of hypothesis to contrast. In this work the absence of hypothesis is a complication for the understanding of the results obtained. In my opinion there would have to be a hypothesis formulation section and subsequently its contrast in the results section or in another subsequent section”* (Reviewer 1).
  - We have divided the Introduction into two sections. The second one is “1.1. Foundations and research hypotheses”. At the end of this section, we have included the following paragraphs:

“Based on the former arguments, we propose the following research hypotheses:

Hypothesis 1 (H_1_): Ethical banking has more economic solvency than conventional banking.

Hypothesis 2 (H_2_): Ethical banking is more linked to the real economy than conventional banking.

Hypothesis 3 (H_3_): Ethical banking is growing more than conventional banking.

In the results section (3.4. Statistical test and 3.5. Probit regressions) we have referenced the validation of these hypotheses.

- *“Authors consider that the financial crisis in 2008 led to a distrust towards conventional banking and, consequently, ethical banking emerged. However, ethical banking came up earlier to cover the needs of certain sectors of the population, specifically the so-called solidarity economy and third sector. Moreover, cooperative and savings banks have been ignored and they were born with similar objectives”* (Reviewer 2).

*“The article only consider two banking models, conventional and ethical banking. However, in several European countries there is also a public banking. Moreover, do cooperative and saving banks (which share characteristics with both types of banking) have to be considered as ethical or conventional banking? Why?* (Reviewer 2).

*“(In the Introduction) The authors use the terms ethical banking and social banking in the same sense, when it is clear that both concepts are different. The difference between them should be specified. This is because cooperative banks and saving banks have been considered as social banks and their differentiation should also be considered”* (Reviewer 3)

*“Ethical banking presents not only a unique model. In effect, Triodos is an anonymous society but the cooperative model (for example, Banca Popolare Etica) has also to be taken into account. This issue has not been mentioned in the paper”* (Reviewer 2).

- In the article, we state that ethical banking was created in the 70s of the 20^th^ century. However, it is after the financial crisis in 2008 when this kind of banking has experienced its main growth. With the objective of being more precise, we added the following paragraph:

“Ethics has been linked to banking since its birth. The first banks arose in Italy in the Renaissance, where Catholicism was the prevailing moral, so that usury was not allowed and the access to credit of most disadvantaged people was favoured. The “Montes de Piedad”, created by religious orders, were the basis for the first saving banks in the first half of the 19^th^ century and had a clear social vocation. Likewise, the cooperative banks, which also emerged in the 19^th^ century, in the midst of the Industrial Revolution, had the mission of financing local projects and favouring the saving of working classes. However, the first ethical investment fund, the Pax World Fund, was created in 1971 on the occasion of the pacifist movement that emerged in the US in the wake of the Vietnam War. People rejected conventional banking to deposit their savings, since it financed arms companies interested in prolonging the conflict. It was in the three last decades of the 20^th^ century when the first properly called ethical banks were created: the South Shore Bank in US (1973), Triodos Bank in Holland (1980), The Cooperative Bank in England (1992), Banca Popolare Etica in Italy (1995) and JAK Medlemsbank in Sweden (1997) (Valls et al. 2019)”.

- In the article, we state that “However, the concept of social banking is wider, since it includes not only ethical banks, but also savings and cooperative banks”. We add the following sentence: “Effectively, savings and cooperative banks share social characteristics with ethical banking and, for this reason, they are jointly included in the concept of social banking, but they are not properly ethical banks, since the whole of their investment is not guided by social-environmental criteria and this is a necessary requirement to be considered ethical banking”. And in the article, it is also said: “Moreover, it is important to differentiate ethical banks from poverty-alleviation banks: the first ones are aimed to gather customer deposits to finance cultural, social and ecological projects, whilst the second ones aim the economic development for the low-income population as well as the community development in marginalized areas, generally through microfinance”.
- Public banking is not the subject of this work. It is a kind of banking very interesting, but it is governed by other different patterns. Its study would be very appealing. However, it escapes our goal and it would require a separate article.
- In this work we have not analysed if a bank has its capital divided into shares or participations. Certainly, it would be a characteristic to study in deep, but we cannot cover all possible areas in a single paper. However, regardless of its corporate form, Banca Popolare Etica has been considered as one of the 13 European ethical banks analysed (see Table 2), although its name is now Banca Etica.
- *“It would also be interesting to provide the bibliographic reference of some previous work that uses the financial economic ratios used in this study”* (Reviewer 1).
  - We have added the following references in Section 2 (Material and methods):
    - Yoshino, N. & Taghizadeh-Hesary, F. (2019). Optimal credit guarantee ratio for small and medium-sized enterprises’ financing: Evidence from Asia. *Economic Analysis and Policy*, 62, 342-356. <https://doi.org/10.1016/j.eap.2018.09.011>
    - Columba, F., Gambacorta, L. & Mistrulli, P.E. (2010). Mutual guarantee institutions and small business finance. *Journal of Financial Stability*, 6(1), 45-54. <https://doi.org/10.1016/j.jfs.2009.12.002>
    - Keister, T. & Morten, L.B. (2012). On the liquidity coverage ratio and monetary policy implementation. *BIS Quarterly Review*, December 2012, 49-61. <https://ssrn.com/abstract=2206362>
    - Rodrigues, L. & Rodrigues, L. (2018). Economic-financial performance of the Brazilian sugarcane energy industry: An empirical evaluation using financial ratio, cluster and discriminant analysis. *Biomass & Bioenergy*, 108, 289-296. <https://doi.org/10.1016/j.biombioe.2017.11.013>
    - Mucharreira, P.R., Antunes, M.G., Abranja, N., Justino, M.R.T. & Quirós, J.T. (2019). The relevance of tourism in financial sustainability of hotels. *European Research on Management and Business Economics*, 25(3), 165-174. <https://doi.org/10.1016/j.iedeen.2019.07.002>
    - Elexa, L., Hvolkova, L. & Knapkova, M. (2019). Anticrisis management: Warning signals before failure. *Marketing and Management Research*, 3, 98-111. <https://doi.org/10.21272/mmi.2019.3-08>
    - Succurro, M., Arcuri, G. & Costanzo, G.D. (2019). A combined approach based on robust PCA to improve bankruptcy forecasting. *Review of Accounting and Finance*, 18(2), 296-320. <https://doi.org/10.1108/RAF-04-2018-0077>
    - Dejuan, O. & Dejuan-Bitria, D. (2018). A predator-prey model to explain cycles in credit-led economies. *Review of Keynesian Economics*, 6(2), 159-179. <https://doi.org/10.4337/roke.2018.02.01>
- *“Authors finish conclusions by stating that “It would be interesting to extent in the future the considered sample to a large number of conventional banks in Europe and even to analyse other continents, as well as a wider period and more variables”. I consider that it would be interesting to include a wide sample of ethical banking in the study of the financial statements and not only Triodos”* (Reviewer 3).
  - We have added the following sentence: “In the future, it would be interesting to include a wide sample of ethical banking in the study of the financial statements, besides Triodos, and to expand the considered sample to a large number of conventional banks in Europe, analyse other continents, as well as a wider period and more variables”.
- “The references Cheong et al. (2016) and Kant et al. (2017) are included in the text, but they are not included in the reference list” (Reviewer 3).
  - Cheong et al. was wrong. The correct reference is “Chew et al.”. The firs author of this article is “Boon Cheong Chew” and it was cited in References as “Chew, B.C….” and this cite is correct. We have corrected this cite in the text.
  - Kant et al. was wrong. The correct is “Khan et al.”. This article was included in References. We have corrected this cite in the text.
- *“I recommend a grammar revision of the text before its possible publication”* (Reviewer 2, Reviewer 3 and Editor).
  - Following your suggestions, we have proof-read the paper.

We will look forward to receiving your final decision.

Best regards,

The authors.

**References**

Columba, F., Gambacorta, L. & Mistrulli, P.E. (2010). Mutual guarantee institutions and small business finance. Journal of Financial Stability, 6(1), 45-54. <https://doi.org/10.1016/j.jfs.2009.12.002>

Dejuan, O. & Dejuan-Bitria, D. (2018). A predator-prey model to explain cycles in credit-led economies. Review of Keynesian Economics, 6(2), 159-179. <https://doi.org/10.4337/roke.2018.02.01>

Elexa, L., Hvolkova, L. & Knapkova, M. (2019). Anticrisis management: Warning signals before failure. Marketing and Management Research, 3, 98-111. <https://doi.org/10.21272/mmi.2019.3-08>

Joecks, J., Pull, K & Vetter K. (2013). Gender Diversity in the Boardroom and Firm Performance: What Exactly Constitutes a “Critical Mass”? *Journal of Business Ethics*, 118, 61-72. <https://doi.org/10.1007/s10551-012-1553-6>

Keister, T. & Morten, L.B. (2012). On the liquidity coverage ratio and monetary policy implementation. BIS Quarterly Review, December 2012, 49-61. <https://ssrn.com/abstract=2206362>

Lin, W.L., Ho, J.A. & Sambasivan, M. (2019). Impact of Corporate Political Activity on the Relationship Between Corporate Social Responsibility and Financial Performance: A Dynamic Panel Data Approach. *Sustainability*, 11(60), 1-22. <https://doi.org/10.3390/su11010060>

Mucharreira, P.R., Antunes, M.G., Abranja, N., Justino, M.R.T. & Quirós, J.T. (2019). The relevance of tourism in financial sustainability of hotels. European Research on Management and Business Economics, 25(3), 165-174. <https://doi.org/10.1016/j.iedeen.2019.07.002>

Reverte, C. (2009). Determinants of Corporate Social Responsibility Disclosure Ratings by Spanish Listed Firms. *Journal of Business Ethics*, 88, 351-366. <https://doi.org/10.1007/s10551-008-9968-9>

Rodrigues, L. & Rodrigues, L. (2018). Economic-financial performance of the Brazilian sugarcane energy industry: An empirical evaluation using financial ratio, cluster and discriminant analysis. Biomass & Bioenergy, 108, 289-296. <https://doi.org/10.1016/j.biombioe.2017.11.013>

Spector, P.E. (2019). Do Not Cross Me: Optimizing the Use of Cross-Sectional Designs. *Journal of Business and Psychology*, 34, 125-137. <https://doi.org/10.1007/s10869-018-09613-8>

Succurro, M., Arcuri, G. & Costanzo, G.D. (2019). A combined approach based on robust PCA to improve bankruptcy forecasting. Review of Accounting and Finance, 18(2), 296-320. <https://doi.org/10.1108/RAF-04-2018-0077>

Valls Martínez, M.C. & Abad Segura, E. (2018). Patient satisfaction in the Spanish National Health System. *Anales del Sistema Sanitario de Navarra*, 41(3), 309-320. <https://doi.org/10.23938/ASSN.0315>

Valls Martínez, M.C., Cruz Rambaud, S. & Parra Oller, I.M. (2019). *La banca ética en España*. In Lena Acebo, F.J. & García Ruíz, M.E. (coord). *Education, Technology, Innovation and Development: Perspectives for a New Era*. Spain: Aula Magna (McGraw-Hill); 2019, pp. 59-69.

Ward, A.M. & Forker, J. (2017). Financial Management Effectiveness and Board Gender Diversity in Member-Governed, Community Financial Institutions. *Journal of Business Ethics*, 141, 351-366. <https://doi.org/10.1007/s10551-015-2699-9>

Yoshino, N. & Taghizadeh-Hesary, F. (2019). Optimal credit guarantee ratio for small and medium-sized enterprises’ financing: Evidence from Asia. Economic Analysis and Policy, 62, 342-356. <https://doi.org/10.1016/j.eap.2018.09.011>
